# Water masses influence the variation of microbial communities in the Yangtze River Estuary and its adjacent waters

**DOI:** 10.3389/fmicb.2024.1367062

**Published:** 2024-03-20

**Authors:** Wen-Dong Xian, Jinhui Chen, Zheng Zheng, Junjie Ding, Yinli Xi, Yiying Zhang, Wu Qu, Chunyu Tang, Changlin Li, Xuezhu Liu, Wei Li, Jianxin Wang

**Affiliations:** ^1^Marine Microorganism Ecological & Application Lab, Marine Science and Technology College, Zhejiang Ocean University, Zhoushan, China; ^2^College of Science, Shantou University, Shantou, China

**Keywords:** Yangtze river estuary, water masses, bacterial diversity, network topology, community assembly

## Abstract

The Yangtze River estuary (YRE) are strongly influenced by the Kuroshio and terrigenous input from rivers, leading to the formation of distinct water masses, however, there remains a limited understanding of the full extent of this influence. Here the variation of water masses and bacterial communities of 58 seawater samples from the YRE and its adjacent waters were investigated. Our findings suggested that there were 5 water masses in the studied area: Black stream (BS), coastal water in the East China Sea (CW), nearshore mixed water (NM), mixed water in the middle and deep layers of the East China Sea (MM), and deep water blocks in the middle of the East China Sea (DM). The CW mass harbors the highest alpha diversity across all layers, whereas the NM mass exhibits higher diversity in the surface layer but lower in the middle layers. *Proteobacteria* was the most abundant taxa in all water masses, apart from that, in the surface layer masses, *Cyanobacterium*, *Bacteroidota*, and *Actinobacteriota* were the highest proportion in CW, while *Bacteroidota* and *Actinobacteriota* were the highest proportion in NM and BS; in the middle layer, *Bacteroidota* and *Actinobacteriota* were dominant phylum in CW and BS masses, but *Cyanobacterium* was main phylum in NM mass; in the bottom layer, *Bacteroidota* and *Actinobacteriota* were the dominant phylum in CW, while *Marininimicrobia* was the dominated phylum in DM and MM masses. Network analysis suggests water masses have obvious influence on community topological characteristics, moreover, community assembly across masses also differ greatly. Taken together, these results emphasized the significant impact of water masses on the bacterial composition, topological characteristics and assembly process, which may provide a theoretical foundation for predicting alterations in microbial communities within estuarine ecosystems under the influence of water masses.

## 1 Introduction

Microorganisms play a crucial role on biochemical cycling and serve as indicators of environmental conditions in marine ecosystems. Microorganisms can survive by gaining energies and nutrients from the geological materials and media (Dong et al., 2022), which including nutrient levels (Arrigo, 2005), oxygenation (Gómez-Plaza and Cano-López, 2011), temperature (Chwastowski et al., 2023), pollution (Rosenberg, 2012), carbon (Soong et al., 2020), nitrogen, carbon, sulfur, iron, and arsenic cycling (Chen et al., 2024). Their abundance and diversity provide valuable insights into nutrient concentrations, oxygen levels, and temperature variations across different oceanic regions (Bianchi et al., 2018). In addition, some microorganisms are particularly sensitive to pollutants, making them useful indicators of ecosystem health (Parmar et al., 2016), some microorganisms may be affected by changes in seawater pH and acidity, making them potential indicators of ocean acidification (Turley and Findlay, 2009a,b). They exhibit substantial abundance, extensive variety, remarkable susceptibility to environmental factors, and always interact with other organisms (Petsch et al., 2001; Torsvik et al., 2002). Additionally, they contribute to regulating marine climate change (Jiao and Azam, 2011) and essential for pollutes converting, nutrient and material circulation of the marine ecosystem, maintaining ecosystem health and alleviating environmental pressure (Zhang W.-C. et al., 2018).

In recent years, there has been a growing scientific interest in understanding the influence of ocean currents in shaping bacterial communities (Skarðhamar et al., 2007; Hsiao et al., 2011). Study by Baltar et al. suggests that hydrological characteristics play an important role in controlling microbial community structure (Baltar and Aristegui, 2017). The hydrological characteristics and distribution of water masses strongly support the idea that the East China Sea (ECS) is a hotspot for marine microbial life, as recently suggested by studies on bacterial, phytoplankton, and zooplankton community compositions (Hsieh et al., 2004; Zhou et al., 2021). The coastal current systems in the Yangtze River estuary (YRE) and its adjacent waters are primarily including the Yangtze River diluted water characterized by low salinity and rich nutrients, as well as the Taiwan warm current (a branch of the Kuroshio) with high salinity but low nutrient content (Li et al., 2014). The Kuroshio also plays a significant role in most hydrodynamic processes in the YRE and its adjacent waters, exerting an important impact on the regional ecological environment, local climate, hydrological conditions, and circulation structure (Guo et al., 2018). According to the conventional perspective (Qi, 2014), The ECS is primarily categorized into three major water systems: the coastal water system, the Kuroshio Water System, and the continental shelf mixed water system., owing to its unique geographical location and complex water systems, the YRE acts as a vital ecological barrier that purifies its flow towards the open sea (Jiang et al., 2017; Yang et al., 2022). The fluctuation process of the water masses in the ECS, the seasonal variation of Taiwan’s warm water, the variability of the Kuroshio flow in the ECS, and the water exchange process on the continental shelf of the ECS influence marine environmental conditions, material transport dynamics, and energy transfer mechanisms within this region. Different water masses would create distinct physical and chemical environments, resulting in differences in microbial communities (Bardgett et al., 2008), however, research on the influence of water masses on the distribution patterns of estuarine microorganisms is limited. Since these ecosystems are typically affected by freshwater outflows and seawater intrusion, it can be hypothesized that the characteristics of the water mass have influences on the diversity of bacteria, species composition, network topology properties, keystone taxa composition, and community aggregation processes in the YRE.

To address the aforementioned hypothesis, we conducted investigations on distribution of water massed and variations of bacterial community based on 58 seawater samples collected from the YRE. 13 environmental factors, Illumina sequencing of bacterial 16S rRNA genes and network analysis were utilized to examine changes in bacterial community following 9 water masses across surface, middle and bottom layers.

## 2 Materials and methods

### 2.1 Sample collection and environmental factor measurement

**Figure 1:**
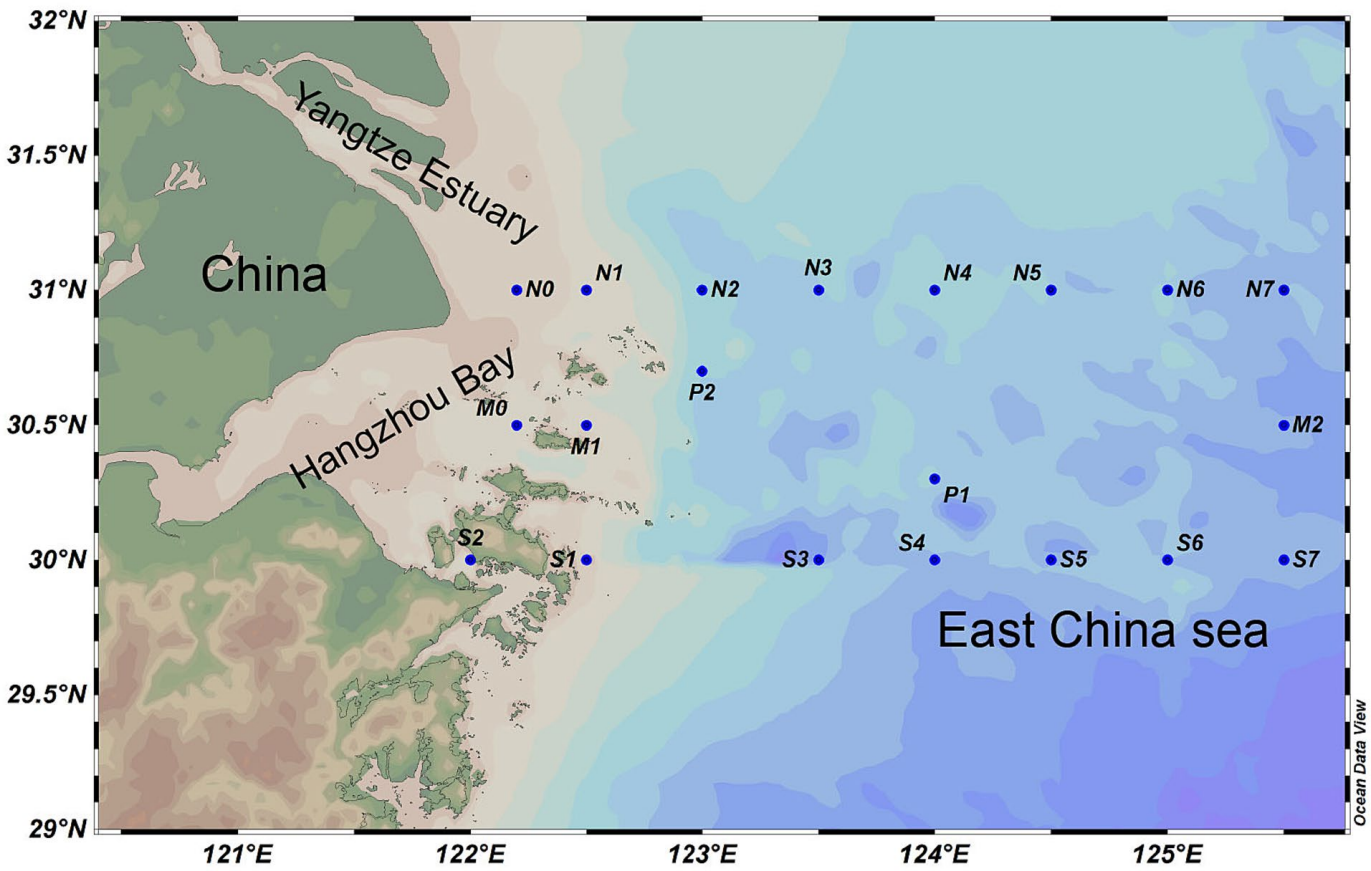
An overview of sampling sites. A total of 58 seawater samples were collected from 23 sampling stations along the YRE. The map was generated with the Ocean Data View (http://odv.awi.de).

Sampling of this study was conducted in August 2021 by the scientific research vessel “Zheyuke 2.” A total of 58 seawater samples from the surface (depth of 1–2 m), middle (depth of 10–35 m), and bottom (within 5 m above the sediment) water columns were collected from 23 sampling stations along the YRE (Figure 1), Only stations with water depths exceeding 20 m were sampled for middle-layer water in this study. All samples were collected using Go-Flo^®^ bottles equipped with conductivity-temperature-depth (CTD) sensors (Sea-Bird Electronics SBE 32). The *in situ* environmental parameters, including temperature, salinity, pH value, and dissolved oxygen (DO), were directly measured using the CTD sensors. The physical and chemical properties including nitrate (NO_3_^−^), nitrite (NO_2_^−^), chemical oxygen demand (COD), silicate (SiO_4_^2−^), phosphate (PO_4_^3−^), total alkalinity (Alk) concentration and chlorophyll a (Chl-a) were determined following the marine monitoring standards of China (Center, N.M.E.M, 2007), Ammonia nitrogen (NH_4_^+^) were analyzed using SmartChem automatic nutrition analyzer (Smartchem 200, Alliance, France). In detail, NO_3_^−^ concentration was measured using copper cadmium column reduction method, the concentration of NO_2_^−^ was measured using diazo coupling method, the potassium permanganate titration method was used to determine the COD concentration, the concentration of SiO_4_^2−^ was measured using the silicon molybdenum blue method, the concentration of PO_4_^3−^ was measured using the phosphorus molybdenum blue method, the total Alk concentration was measured using the pH potential drop method, the concentration of Chl a was determined using fluorescence spectrophotometry.

### 2.2 DNA extraction, sequencing, and amplicon analysis

Total DNA of each sample was extracted by the PowerSoil DNA isolation Kit (Mo bio, San Diego, CA, United States) according to the instructions. The quality of DNA was evaluated by 1% agarose gel electrophoresis and nanodrop 2000 (Thermo, Waltham, Ma, United States). The v4–v5 regions of the 16S rRNA gene were amplified using the primers 515F (5′-GTGCCAGCMGCGG-3′) and 907R (5′-CCGTCAATTCMTTRAGTTT-3′) (PCR instrument: abigeneamp^®^ Model 9,700). The PCR products were purified with AxyPrepDNA gel recovery kit (Aisijin Biotechnology Co., Ltd.) for library construction, sequencing libraries were generated using Illumina HiSeq Platform, the library quality was assessed on NCBI database, high-throughput sequencing was performed by Majorbio Pharm Technology Co., Ltd. (Shanghai, China) on the Illumina miseq platform.

FLASH (fast length adjustment of short reads) was used to merge the original two terminal sequences (Chen et al., 2018). The original sequences of the paired-end sequencing were processed using the QIIME2 platform (Hall and Beiko, 2018). Primer excision and quality control were performed using vsearch (Rognes et al., 2016). Sequence analyses were performed by QIIME2 with open reference_otus.py, sequences with 97% similarity were assigned to the same ASVs (Caporaso et al., 2010). The SILVA database (v138) (Quast et al., 2012) was used to taxonomic annotation of each ASV based on the RDP classifier (Lan et al., 2012). The ASVs table was generated by mapping the primer removed reads to the representative sequences of ASVs.

### 2.3 Statistical analysis

The water masses in the continental shelf region usually tend to gather according to variations in both water temperature and salinity (Qiao et al., 2006; Li et al., 2018). Therefore, the “fpc” package in R was utilized to cluster the 58 samples into different groups based on their temperature and salinity (Li et al., 2018). Several packages in R software (Zhang et al., 2019) have been adopted to perform statistical analyses in this study. The “vegan” package (Edgar, 2010) was used for resampling, the “ggplot2” package (Oksanen et al., 2013) was used to generate all figures. “corrplot R” (Wei et al., 2017) package for calculating coefficients and the “picant” package (Wang et al., 2021) for estimation of diversity indices. The Wilcoxon test was used to check community variations between different groups in each water layer. The Bray Curtis distances were calculated using the package “vegan” (Edgar, 2010) with the relative counts of ASVs, and visualized using non metric multidimensional scale analysis (NMDS). Linear multivariate redundancy analysis (RDA) was used to analyze the correlation between bacterial community structure and environmental factors at the ASV level. Community composition maps were visualized using the package of “statnet” (Abdi and Williams, 2010) and “circlize” (Gu et al., 2014). To construct the ecological network of microbial community, we utilized the Conet algorithm in Cytoscape software (v3.9.1). Furthermore, we employed the “rnetcarto” (Doulcier and Stouffer, 2015) package to evaluate the intra-module connectivity (Zi) and inter-module connectivity (Pi) of microbial species, thus enabling the identification of keystone taxa within each community. Neutral models explore microbial taxa abundance through stochastic dispersal, random species formation, and ecological drift (Chen et al., 2019). *R*^2^ indicates overall goodness of fit, while *m*-values represent community-level mobility. *Nm*-values result from multiplying metacommunity size (*N*) by *m*-values. They are used to analyze changes in community composition patterns visually (Zhou et al., 2010a,b).

## 3 Result

### 3.1 Identification of water masses

**Figure 2:**
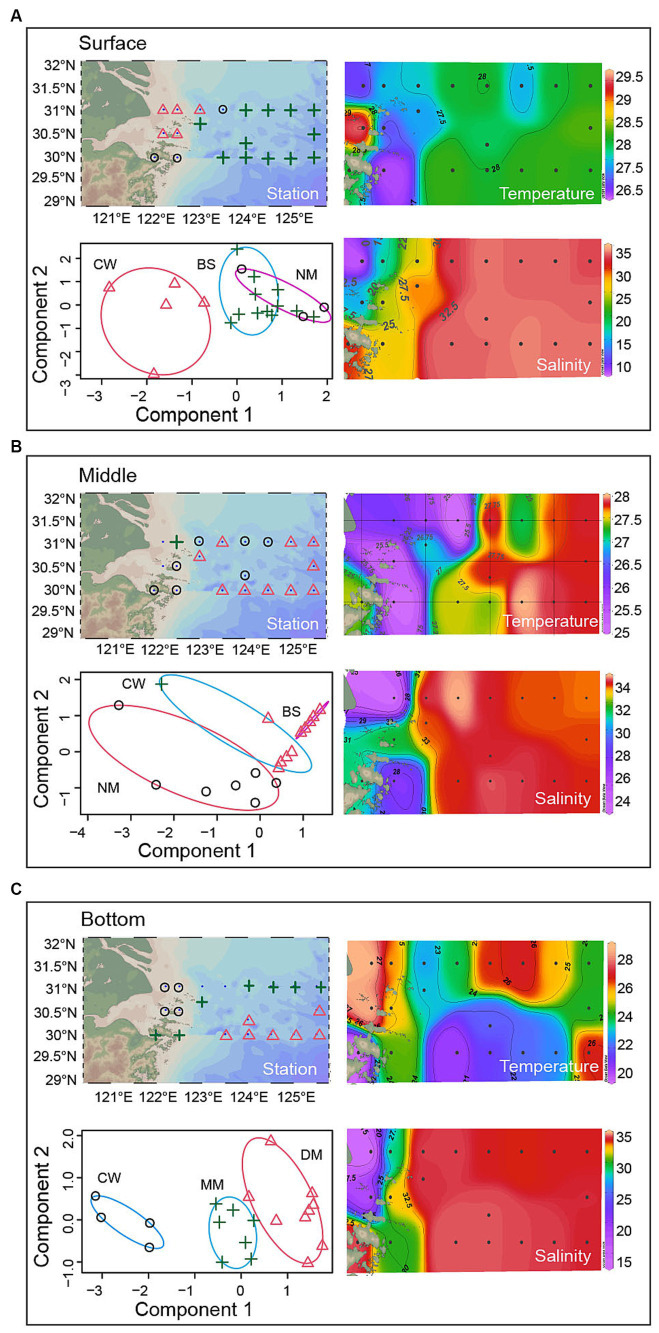
The distribution of water masses across the surface, middle and bottom layers.

Samples of surface (20 water samples), middle (20 water samples) and bottom (18 water samples) seawater layers were divided into three categories, respectively (Figure 2). Then, to identify these categories of water sample, the water characteristics was compared with a previous study (Table 1) (Pan et al., 2016). The results showed that the study area can be divided into Black Stream (BS) water mass, coastal water in the East China Sea (CW) water mass, nearshore mixed water (NM) water mass, middle deep mixed water in the East China Sea (MM) water mass, deep-water mass in the middle of East China Sea (DM). Finally, in the surface layer, M0, M1, N0, N1, and N2 were identified as CW mass (T: 26.80°C–29.60°C, S: 7.68–24.93 psu). M2, N4, N5, N6, N7, P1, P2, S3, S4, S5, S6, and S7 were identified as BS mass (T: 27.42°C–28.23°C, S: 32.63–33.53 psu). N3, S1, and S2 were identified as NM mass (T: 26.59°C–27.99°C, S: 25.61–32.48 psu). In the middle layer, N1 were identified as CW mass (T: 26.11°C, S: 24.96 psu). M2, N3, N6, N7, P2, S3, S4, S5, S6, and S7 were identified as NM mass (T: 25.31°C–28.01°C, S: 31.17–33.61 psu). M1, N2, N4, N5, P1, S1, and S2 were identified as BS mass (T: 25.25–28.01°C, S: 27.84–33.61 psu). In the bottom layer, N0, M0, N1, and M1 were identified as CW mass (T: 24.91°C–27.78°C, S: 13.62–32.52 psu). M2, N2, N3, P1, S3, S4, S5, S6, and S7 also were identified as CW mass (T: 20.85°C–26.08°C, S: 20.85°C–36.08°C, S: 20.62–32.52 psu). N2, N3, P1, S3, S4, S5, S6, and S7 were identified as DM mass (T: 20.85°C–26.08°C, S: 33.81–34.30 psu). N5, N6, N7, P2, S1, S2, and N4 were identified as MM mass (T: 20.03°C–26.20°C, S: 29.95–34.24 psu).

**Table 1:** Characteristic values of water masses in the YRE and its adjacent waters (Pan et al., 2016).

Water masses	Temperature/°C	Salinity/PSU	Depth/m
coastal water in the East China Sea (CW)	26–28	10–30	0–10
Nearshore mixed water (NM)	25.4–27.6	30.6–32.8	0–20
Black stream (BS)	26.1–29.3	32.3–33.7	0–18
Deep water mass in the middle of the East China Sea (DM)	19.0–20.8	34.3–34.5	30-seabed
mixed water in the middle and deep of the East China Sea (MM)	21.9–22.8	34–34.1	30-seabed

### 3.2 Bacterial diversity

**Figure 3:**
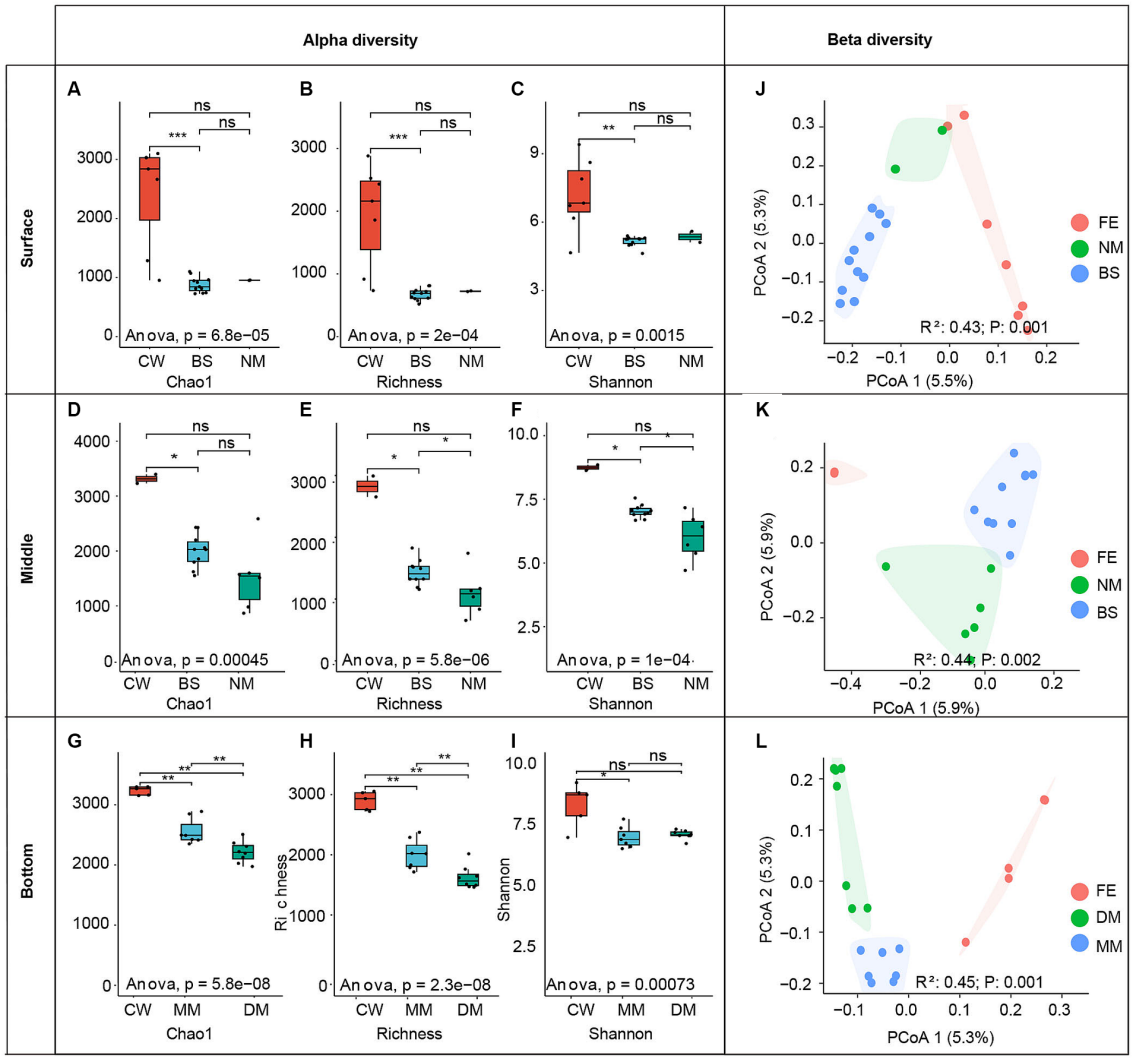
Left panel: alpha diversity indexes of different water layers (B, M, S). The group differences of Chao1 index **(A)**, richness index **(B)**, and Shannon index **(C)** of different water masses in surface **(A–C)**, meddle **(D–F)**, and bottom **(G–I)** seawater layers. Asterisks indicate the significance of the difference between the two groups (“***” indicates that the *p*-value is less than 0.001; “**” indicates that the *p*-value range is 0.001–0.01; “*” indicates that the *p*-value range is 0.01–0.05, “ns” indicates that the *p*-value is greater than 0.05). Right panel: principal coordinate analysis (PCoA) of bacterial communities based on Mountfort distance.

The alpha diversity indices, including Chao1, Shannon index and ASVs Richness, were calculated among water masses (Figures 3A–I). The results indicate that, all three indices of CW mass community were highest across three layers. Additionally, significant differences (*p* < 0.01) in the Chao1, Shannon, and Richness index were observed between CW and BS mass in the surface seawater (Figures 3A–C). In the middle layer, differences (*p* < 0.05) were also observed among the Chao1, Shannon and Richness index between CW and BS mass (Figures 3D–F), moreover, the Shannon index and Richness index exhibit differences (*p* < 0.05) between BS and NM. In the bottom layer, significant differences (*p* < 0.05) of Chao1 and Richness index were observed across all water masses, the significant difference (*p* < 0.05) of Shannon was also observed between CW and DM mass community (Figures 3G–I).

To measure the differences in bacterial composition among samples, the beta diversity index was analyzed. The PCoA analysis based on the Mountford distance indicates a significant difference (*R*^2^ = 0.43–0.45; *p* < 0.05) between bacterial communities among water masses within the same water layer (Figures 3J–L). In detail, the ANOSIM analysis revealed significant differences (*R*^2^ = 0.43, *p* = 0.005) among CW, NM and BS masses in the surface layer (S-CW, S-NM, S-BS). Similarly, significant differences (*R*^2^ = 0.44, *p* < 0.005) were observed among BS, CW, and NM masses in the middle layer (M-S, M-FE, M-M). Additionally, there were significant differences (*R*^2^ = 0.45, *p* < 0.001) among MM, DM and CW masses in the bottom layer (M-MM, M-DM, M-CW).

**Figure 4:**
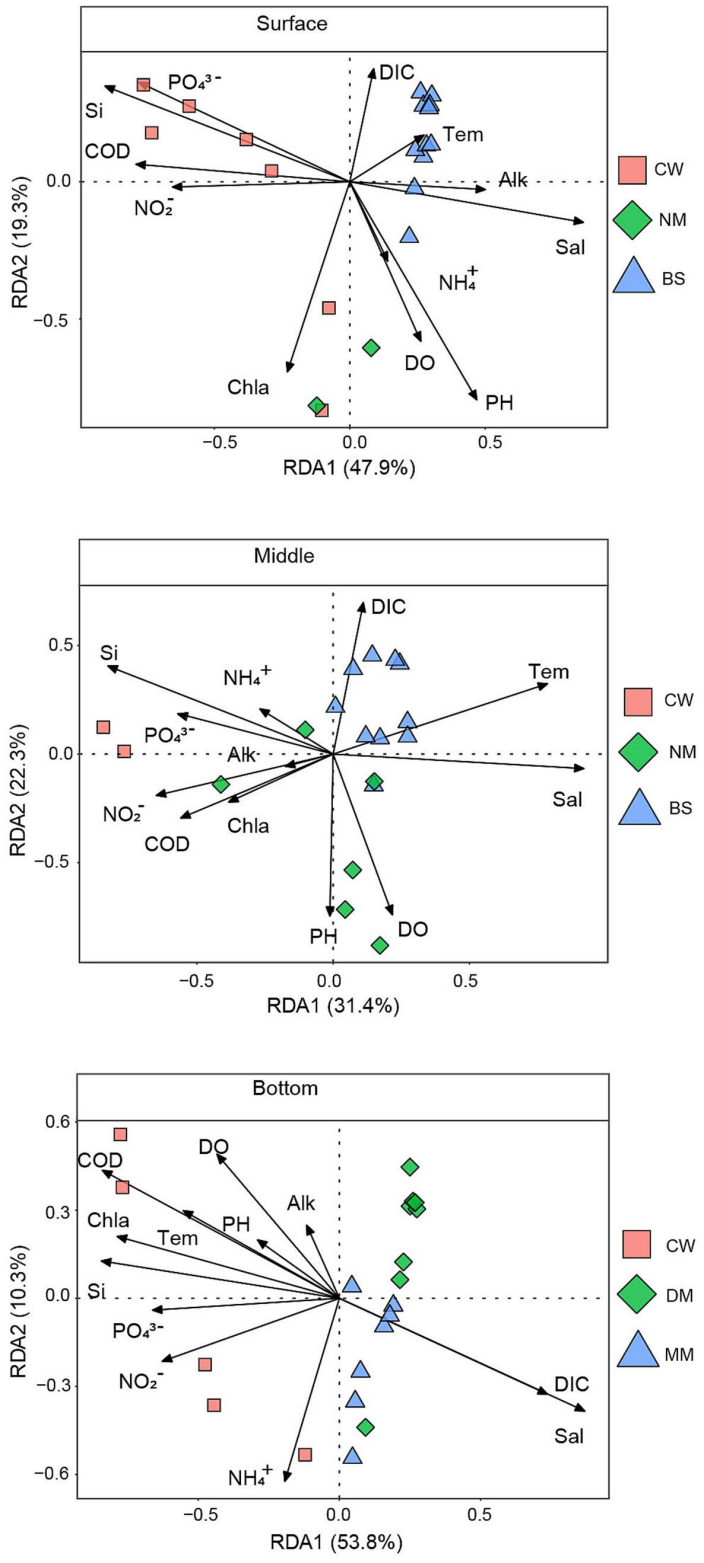
The relationship between environmental factors and the bacterial community.

We conducted redundancy analysis (RDA) to understand the relationship between microbial communities and environmental factors (Figure 4). The RDA results explained 67.2%, 53.7% and 64.1% of the variation in the surface, middle and bottom bacterial communities, respectively. The Mantel test revealed significant correlations between microbial communities in different layers and water masses with 13 environmental variables (Supplementary Table S2). The results indicated that COD, Chl a and NO_2_^−^ were primary environmental factors significantly affecting community structure (*p* < 0.05). In the surface layer (Supplementary Table S2). In the middle layer, salinity, DO and temperature were identified as important environmental factors affecting community structure as well (*p* < 0.05) (Supplementary Table S2). In the bottom layer, COD, DO, Chl a, PO_4_^3−^, temperature, salinity, DIC were the main environmental factors affecting community structure (*p* < 0.01) (Supplementary Table S2).

### 3.3 Bacterial community composition across water masses

**Figure 5:**
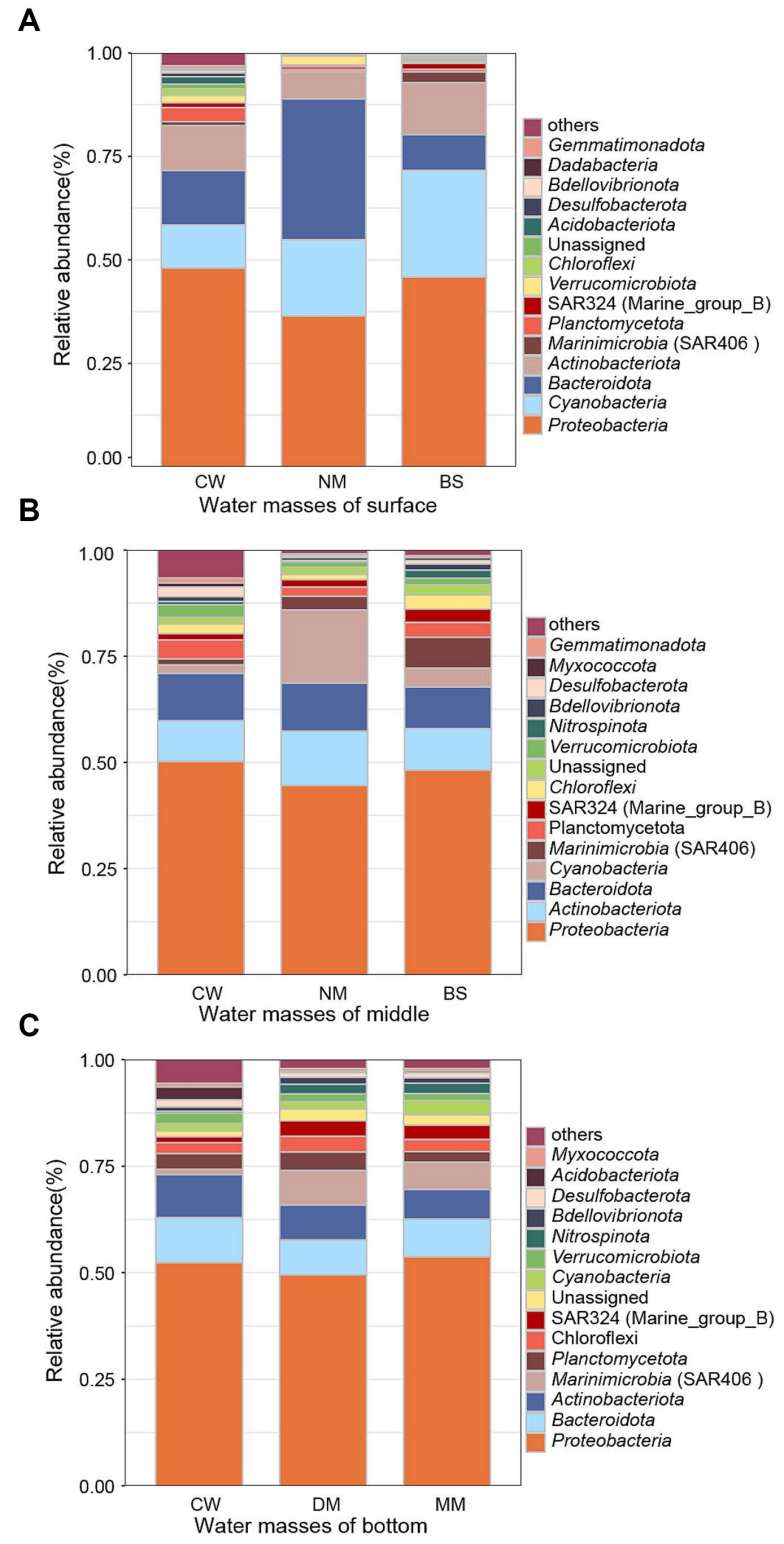
Bacterial community composition. The relative abundances of dominant bacterial phyla in surface **(A)**, middle **(B)**, and bottom **(C)** seawater community bacteria samples. Others indicate the other phyla except the top 15 in relative abundance in the total samples. BS (Black stream) water mass, CW (coastal water of the East China Sea) water mass, NM (nearshore mixed water) water mass, MM (middle deep mixed water in the East China Sea) water mass, DM (deep-water mass in the middle of the East China Sea) water mass.

The distribution of bacterial phyla of different water masses at the surface, middle, and bottom layers were analyzed (Figure 5). *Proteobacteria* was detected as the dominant bacterial phylum across all nine water masses. In the surface seawater, *Cyanobacteria* exhibits the highest proportion in BS (25.73%), followed by NM (18.4%) and CW (10.44%), alongside *Bacteroidota* showing the highest proportion (34.05%) in NM, followed by CW (13.16%) and BS (8.69%). Notably, *Actinobacteriota* demonstrates varying proportions across 3 water masses, with BS having the highest proportion (12.56%). In contrast, CW displays higher proportions (15.05%) of other bacterial phyla compared to BS (7%) and NM (4.49%) (Figure 5A). In the middle layer, *Actinobacteriota* emerges as the second most abundant phylum, *Actinobacteriota* was found in all 3 water masses, with NM having the highest proportion (12.78%), followed by BS (9.81%) and CW (9.59%). The proportion of *Cyanobacteria* varies across CW, with CW showing a lower proportion (2%) compared to the NM (17.23%) and BS (4.41%), while the proportion of *Planctomycota* in CW (4.44%) was higher than NM (2.12%) and BS (3.5%), (Figure 5B). In the bottom seawater, the bacterial phyla with high abundance were *Bacteroidota*, *Actinobacteriota*, and *Marininimicrobia* (SAR406_clade). In CW, however, the proportion (1.41%) of *Marininimicrobia* (SAR406_clade) was lower compared to NM (3.2%) and BS (7.27%) (Figure 5C).

### 3.4 Topological characteristics and keystone taxa of bacteria across water masses

**Figure 6:**
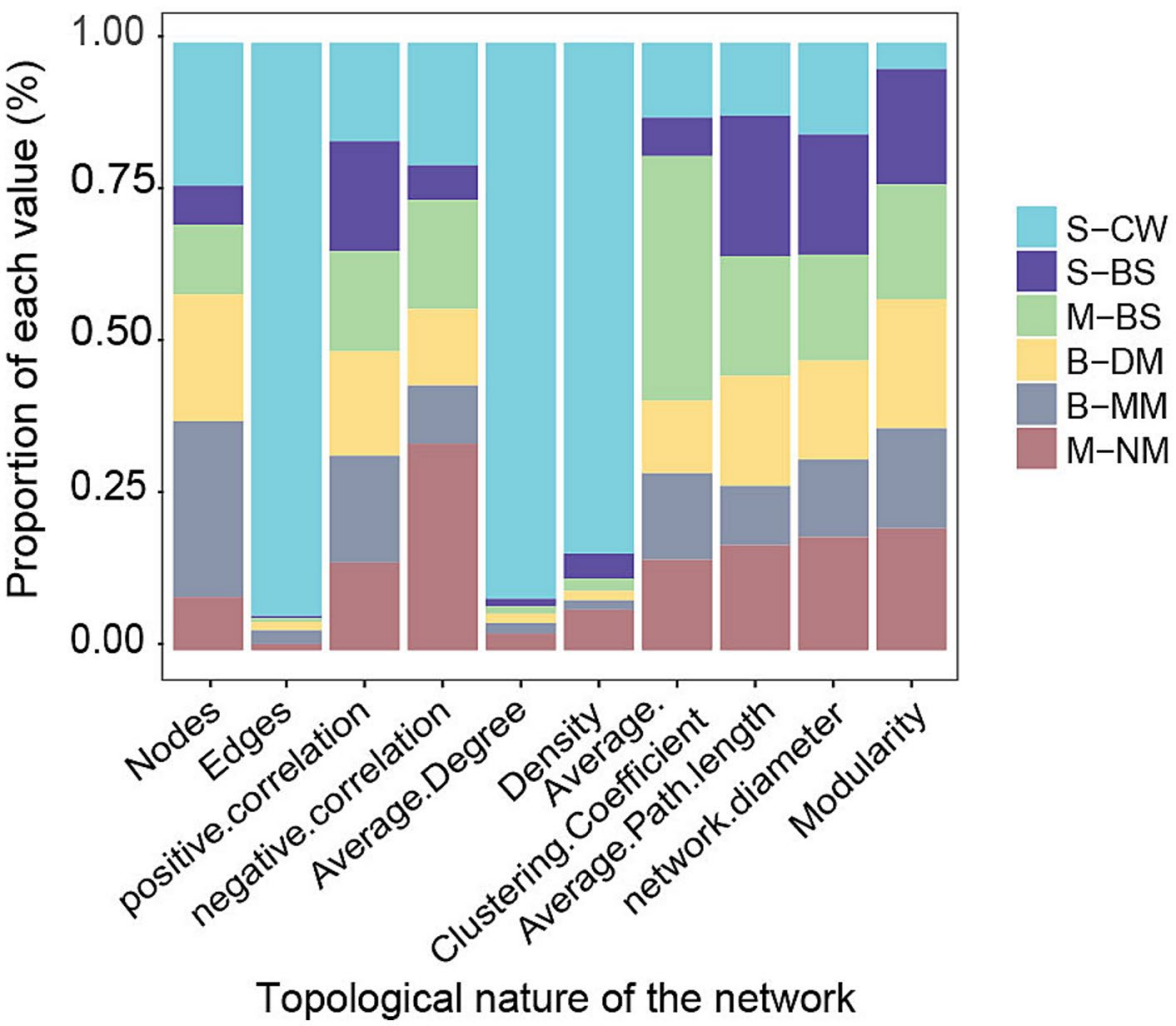
Coexistence network indicators of different water masses in the YRE and its adjacent waters.

To compare the topological characteristics of microbial communities across different water masses, the ecological network was calculated (Table 2). The results of the topological properties were plotted in a columnar stacked graph (Figure 6). The nodes (1121), edges (102948), average degree (183.67), and density (0.16) of network in the S-CW were higher compared to those in other water masses. Moreover, the average path length is lower than that of other water masses. These findings indicate that the network size of this water mass is larger than other masses, with lower separation and higher connectivity. Apart from S-CW, the nodes and edges in the B-MM exhibit highest values compared to other water masses, indicating a larger network size in the B-MM. The average clustering coefficient and average path length in the M-BS were also higher than those of other water masses, suggesting lower separation and greater connectivity. Furthermore, the correlations between nodes across different water masses were predominantly characterized by positive correlations (76.66–96.1%).

**Table 2:** Coexistence network indicators of different water masses in the YRE and its adjacent waters.

Topological parameter	S-CW	S-BS	M-BS	B-DM	B-MM	M-NM
Nodes	1,121	305	546	995	1,378	417
Edges	102,948	388	645	1,534	2,432	1,140
positive correlation %	86.15	96.1	87.68	91.35	93.43	76.66
negative correlation %	13.85	3.9	12.32	8.65	6.57	23.34
Average degree	183.67	2.54	2.36	3.08	3.53	5.47
Density	0.16	0.01	0.01	0.01	0.01	0.01
Average clustering coefficient	0.69	0.36	2.27	0.68	0.80	0.84
Average path length	3.16	6.08	5.18	4.77	2.56	4.55
Network diameter	13	17	15	14	11	16
Modularity	0.20	0.87	0.86	0.97	0.75	0.92

S-CW, the surface layer (S) of coastal water mass in the East China Sea (CW); S-BS, the surface layer (S) of Black stream water mass (BS); M-BS, the surface layer (S) of Black stream water mass (BS); B-MD, the bottom layer (B) of deep water mass in the middle of the East China Sea mass (DM); B-MM, the bottom layer (B) of middle deep mixed water mass in the East China Sea (MM); M-NM, the middle layer (M) of nearshore mixed water mass (NM).

**Figure 7:**
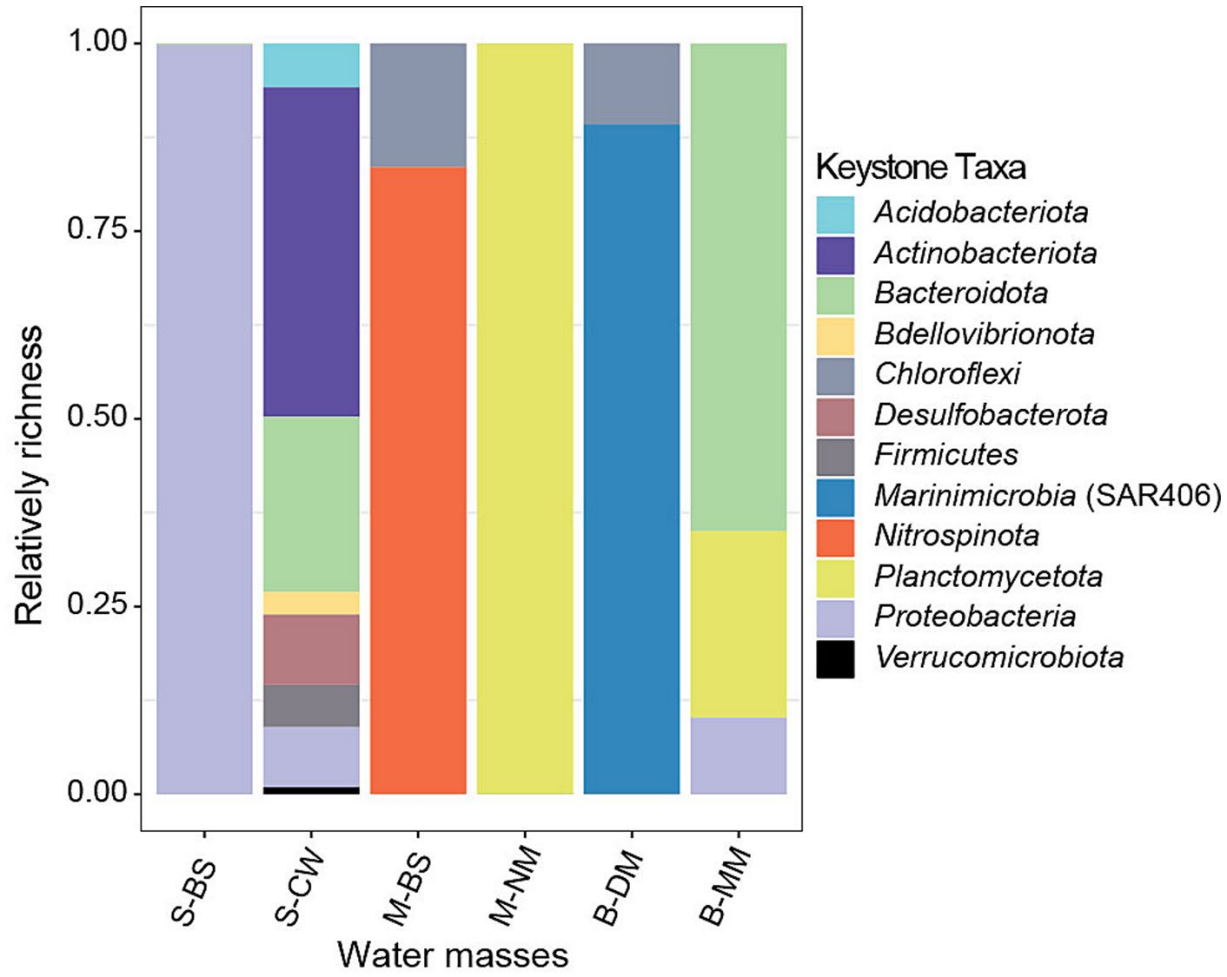
Keystone taxa composition of bacterial community.

In co-occurrence networks, different nodes represent various microbial species, and key species of the community can be identified based on the topological characteristics of the nodes. Node attribute types are typically classified into four categories: peripheral nodes (Zi < 2.5, Pi < 0.62), connector nodes (Zi < 2.5, Pi ≥ 0.62), module hub nodes (Zi ≥ 2.5, Pi < 0.62), and network hub nodes (Zi ≥ 2.5, Pi ≥ 0.62). According to previous research (Cox et al., 2016), nodes with Zi ≥ 2.5 or Pi ≥ 0.62 are defined as key species, based on their intra-module connectivity (Zi) and inter-module connectivity (Pi) in the network. Generally, all three types of nodes, except for peripherals, are classified as key nodes (Deng et al., 2012). As depicted in Supplementary Figure S1, it was evident that S-CW exhibits the highest number of connectors, indicating a closer interaction between network modules in this water mass. Furthermore, there is only one modular hub in S-BS, M-BS, B-MM, and B-DM, whereas no modular hub was observed in S-CW and S-NM. This suggests a high degree of interconnections between species within each water mass. Figure 7 illustrates the composition of key species in bacterial communities at the phylum level across six water masses. The species composition of key species in S-CW was relatively complex, with the majority belonging to *Actinobacteriota* and *Bacteroidota* phyla. The key species in M-BS predominantly belong to *Nitrospinota*, while those in M-NM were mainly represented by *Planctomycota*. In B-DM, the majority of key species belong to *Marininimicrobia* (SAR406_clade), whereas most of the key species in B-MM belong to *Bacteroidota*.

### 3.5 Analysis of bacterial assembly process

**Figure 8:**
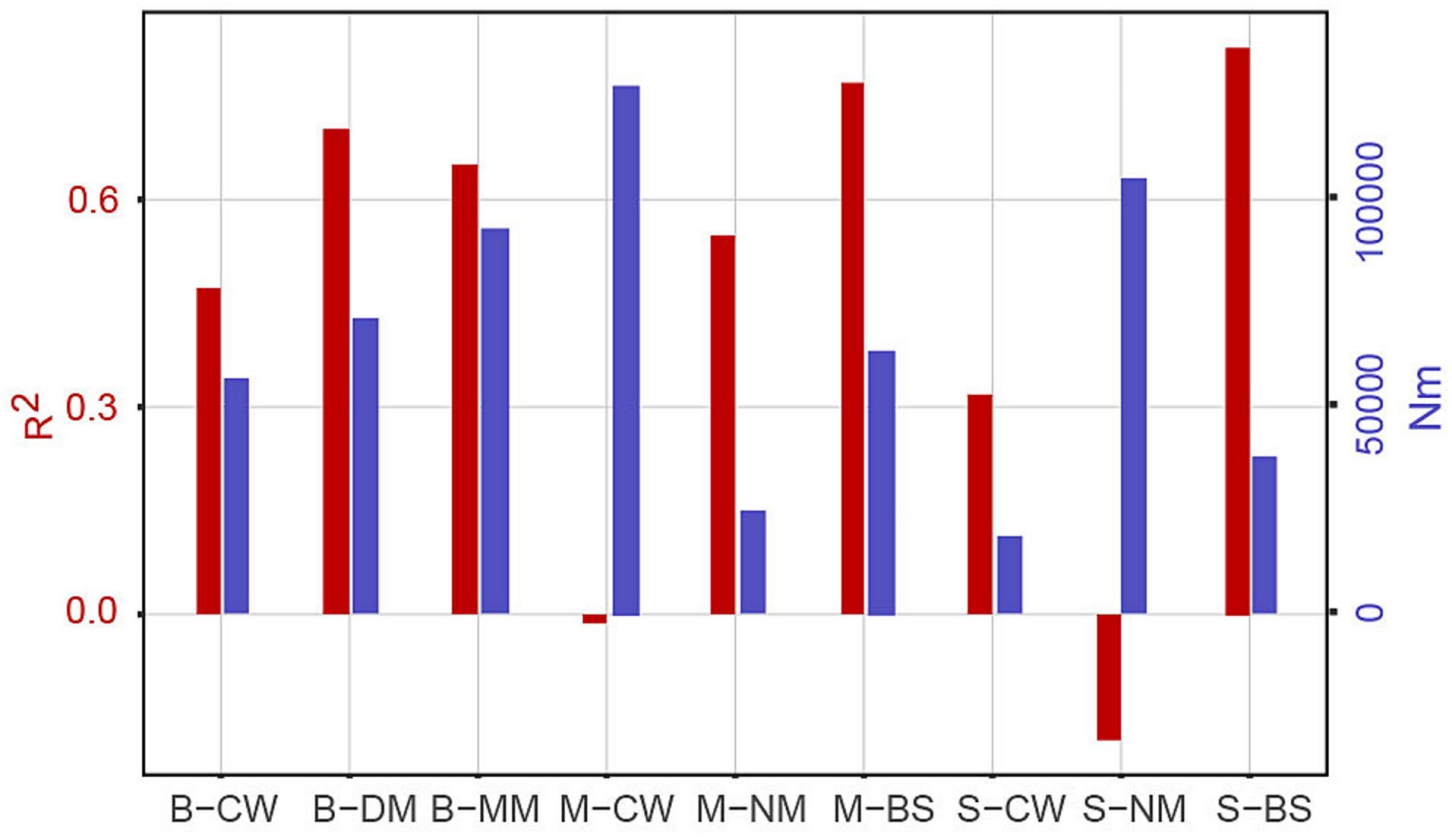
The neutral community model. Rsqr represents overall goodness of fit, *m*-value quantifies community level migration rate, and *Nm* value is the product of metacommunity size (*N*) and *m*-value. “Rsqr” commonly refers to *R*-squared, which is a statistical measure used to assess the goodness-of-fit of a regression equation to the observed data. *n* community ecology, “Nm” typically refers to the migration rate or population dispersal rate within a neutral model.

The NCM analysis successfully captured a significant portion of the relationship between the occurrence frequency of ASVs and their mean relative abundances (Supplementary Figure S2; Figure 8). The results indicate that, compared to other water masses, the impact of stochastic processes has a greater influence on B-MM, M-CW, and S-NM, while M-NM and S-CW and S-BS were more influenced by deterministic processes. Additionally, species diffusion within communities in B-CW and M-BS was more restricted compared to other water masses, whereas species in B-DM experience less constraint on diffusion.

## 4 Discussion

Water masses are commonly occurring in the estuarine environment, yet little is known about the influences on microbial community across the YRE ecosystems. In this study, we analyzed the bacterial diversity in various water masses occurred in the YRE and its adjacent waters. Across the studied area, five distinct water masses were identified. Significant differences of bacterial communities were observed when considering water masses on layers at different depths (Figures 3J–L), the great influence of dramatic changes in environmental gradients in a same water mass resulted in no apparent differences across all masses (Supplementary Figure S3), as a result, the communities in a mass were analyzed in surface, middle and bottom water layers.

### 4.1 Bacterial diversity and dominant taxa among water masses

The coastal current system in the YRE is primarily composed of Kuroshio, coastal, and continental shelf mixed water masses (Song, 2011). The Taiwan warm water mass, a branch of the Kuroshio, flows northward through the Taiwan Strait to the ECS, leading to an increase in both temperature and salinity within the sea water (Jan et al., 2002). The significant higher microbial diversity in CW water mass (Figure 3) may be attributed to warm temperature from Taiwan warm water mass and richer nutrients from coastal seawaters (Margesin et al., 2017). *Proteobacteria* was the dominant phylum in water masses at all layers, which consistent with previous results obtained from pure cultures in the YRE (He et al., 2013). In the surface seawater, except for *Proteobacteria* and *Cyanobacteria* in BS mass, *Bacteroidota* occupies the highest proportion compared to the other two water masses, followed by CW and NM. Freshwater from the Yangtze River influences the salinity and temperature (Ferrenberg et al., 2013), which may lead to a relatively low proportion of *Cyanobacteria* in the surface seawater masses. In comparison, the distribution of *Actinobacteriota* varied across water masses, with CW exhibiting the highest proportion. This observation is consistent with previous research (Yang et al., 2023), which indicates a negative correlation between salinity levels and the abundance of *Actinobacteriota*. In the middle seawater, there was a significant difference in the proportion of *Cyanobacteria* among the various water masses. Specifically, the proportion of *Cyanobacteria* in CW was lower than that in the other two water masses, this finding contradicts previous research results, which claimed that *Cyanobacteria* were more likely to thrive in water with lower salinity (Śliwińska-Wilczewska et al., 2019). In the bottom seawater, *Bacteroidota*, *Actinobacteriota* and *Marinimicrobia* (SAR406_clade) account for a relatively high abundance, the proportion of *Marinimicrobia* (SAR406_clade) in CW was lower than that in the other two water masses, which is consistent with the previous study of the Pearl River estuary (Sang et al., 2022).

Temperature and salinity as crucial factors impacting the aggregation of bacterial communities in oceanic environments (Gilbert et al., 2009). Our findings with the RDA and Mantel analyses further revealed that salinity, temperature, COD, Chl a, and NO_2_^−^ significantly influence the bacterial communities in the seawater masses. These findings suggests NO_2_^−^ is another important environmental factor influencing bacterial diversities among water masses in the YRE.

### 4.2 Topological characteristics of networks and keystone taxa within different water masses

Network topological characteristics play the crucial role in evaluating the health of ecosystems and identifying potential targets for biotechnological interventions (Bull et al., 2000). In this study, the S-NM, M-CW, B-CW were omitted due to the limited samples. The correlations among microbes in different water masses were primarily characterized by positive relationship (Figure 8). However, the negative correlation ratio of M-NM and S-BS was slightly higher compared to other water masses in different layers, this suggests that the competition between species within these two water masses was greater than in other water masses. The S-CW and B-MM exhibit higher network connectivity, which indicates communities in these water mass possesses more complex inter-species interactions in comparison to other water masses. The bacterial networks within S-CW (Density = 0.164) and M-NM (Density = 0.013) were denser than other water masses, which suggest the more complex interactions, greater stability, and stronger stress resistance microbial community harbors in these water mass.

Among the different water masses, S-CW and B-MM exhibit relatively high network average and average clustering coefficients, as well as shorter average paths. These findings suggest that microbial communities residing within these two water masses possess enhanced connectivity and participate in more complex species interactions (Zhang B. et al., 2018; Yang et al., 2019). Therefore, the microbial communities in the S-CW and B-MM closely interact with each other and respond rapidly to external disturbances, but the community exhibits less stability. Conversely, the microbial communities in the middle and bottom seawater masses demonstrate a buffering effect on external disturbances, resulting in strong community stability. Furthermore, the bacterial community network in the S-CW and B-MM was more complex, indicating a closer association between the microbes (Yang et al., 2019). Additionally, the modularity parameters of the network diagrams in all water masses (except for CW) in this study were found to be greater than 0.4, This indicates that the overall topology of network in S-BS, M-BS, B-DM, B-MM, and M-NM exhibits a high level of modularity (Qin et al., 2021). Similar to other complex environments, such as networks constructed with soil (Williams et al., 2014), wetland sediments (Huang et al., 2019).

Keystone taxa are nodes that exhibit a high degree of connectivity within a network (Xian et al., 2020), these particular species hold a unique position within a microbial community, the absence of them impacts both the structure and function of microbial community (Modlmeier et al., 2014). In this study, keystone taxa mainly distributed in *Marinimicrobia*, *Planctomycetota*, *Bacteroidota*, *Nitrospinota*, and *Proteobacteria*. *Planctomycetota*, *Bacteroidetes*, the anaerobic planktonic bacteria within aquatic environments, serves as the main member responsible for the degradation of organic matter in the water column (Sun et al., 2021). Interestingly, *Proteobacteria* was the dominant phylum of in all water masses, but not the primary keystone taxa at phylum across water masses, and only identified as keystone taxa at phylum in three masses (S-BS, S-CW, and B-MM). The possible explanation was that the keystone taxa always not the dominant group (Cupit et al., 2019).

### 4.3 Assembly process of bacterial communities in different water masses

The neutral community model is commonly employed to assess the impacts of random diffusion and ecological drift on bacterial community assembly. In this model, *R*^2^ represents the overall goodness of fit, *m*-value quantifies the community-level migration rate, and *Nm* value is the product of metacommunity size (*N*) and *m*-value. The *m*-value reflects the migration rate at the community level. A higher *m*-value indicates less restrictions on species diffusion within the entire community, while a lower *m*-value suggests higher constraint on species dispersal (Su et al., 2022). A higher *R*^2^ value indicates a closer fit to the neutral model, suggesting that community assembly is predominantly influenced by stochastic processes rather than deterministic processes (Sloan et al., 2006). Using the neutral community model, we evaluated the differences in bacterial community assembly processes across various water masses within the YRE and adjacent environments.

The previous study conducted in the YRE claimed that stochastic processes dominate the assembly of bacterial communities (Shi et al., 2023). Despite the similar conclusion of B-MM and M-CW, the *Nm* value of M-NM, S-CW, and S-BS were less than 5,000 (the highest value in B-MM was 92,918) (Supplementary Figure S2), suggesting bacterial assembly process was not directly controlled by geographical niches in this zone. Instead, water mass dynamics strongly affect the assembly process of microorganisms in the same area. The YRE is eutrophic, and the availability of nutrients for planktonic microbes makes it compatible with nutrient-rich substrates, Besides the temperature and salt, differences of dissolved organic matter (Guo et al., 2018) and nutrient substrates (Catão et al., 2021) among masses may also influences the assembly process. Moreover, there exist unmeasured variables or factors that could potentially impact these dynamics, necessitating further investigation to elucidate their influence (Jiao and Lu, 2020).

## 5 Conclusion

This study investigated the effects of water masses on the variation of bacterial community in the YRE. The surveyed sea area was divided into five distinct water masses: Black Stream (BS) water mass, coastal water in the East China Sea (CW) water mass, nearshore mixed water (NM) water mass, middle deep mixed water in the East China Sea (MM) water mass, deep-water mass in the middle of the East China Sea (DM). In the surface layer, *Cyanobacteria* and *Bacteroidota* were the dominant phylum in BS water mass, while *Actinobacteriota* has the highest proportion in CW. In the middle seawater, the proportion of *Cyanobacteria* in CW was lower than that of the other two water masses, whereas the proportion of *Planctomycota* was higher. Additionally, the proportion of *Marininimicrobia* (SAR406_clade) in CW was lower than in the other two water masses. In the bottom seawater layer, the bacterial phyla with high abundance were *Bacteroidota*, *Actinobacteriota*, and *Marininimicrobia*. In CW, however, the proportion (1.41%) of *Marininimicrobia* (SAR406_clade) was lower compared to NM (3.2%) and BS (7.27%). The network analysis results suggested the interaction among bacterial communities in distinct water masses were primarily characterized by positive correlation. Both stochastic and deterministic processes influenced bacterial assembly processes among the diverse water masses.

## Data availability statement

The datasets presented in this study can be found in online repositories. The names of the repository/repositories and accession number(s) can be found at: https://www.ncbi.nlm.nih.gov/sra/?term=PRJNA1000040.

## Ethics statement

This article does not contain any studies involving to human participants or animals. Sample collection is following local regulations and is approved by management.

## Author contributions

W-DX: Supervision, Validation, Writing – original draft, Writing – review & editing. JC: Investigation, Software, Writing – original draft. ZZ: Data curation, Investigation, Writing – review & editing. JD: Investigation, Software, Writing – review & editing. YX: Investigation, Writing – review & editing. YZ: Investigation, Methodology, Writing – review & editing. WQ: Methodology, Software, Writing – review & editing. CT: Methodology, Software, Writing – review & editing. CL: Writing – review & editing. XL: Data curation, Writing – review & editing. WL: Writing – review & editing. JW: Funding acquisition, Writing – review & editing, Conceptualization.
