# Supplementary figures and images for: Water masses influence the variation of microbial communities in the Yangtze River Estuary and its adjacent waters
Source: Front Microbiol. 2024 Mar 20;15:1367062. doi: 10.3389/fmicb.2024.1367062 (PMC10987813; doi:10.3389/fmicb.2024.1367062)

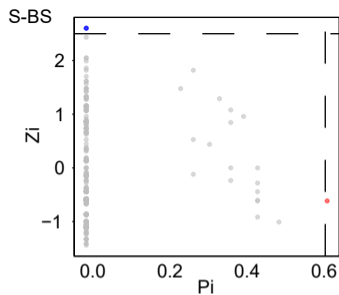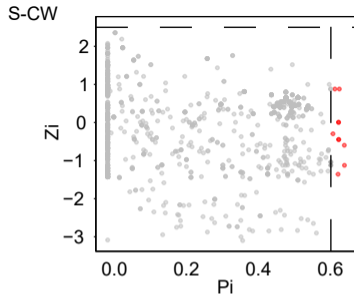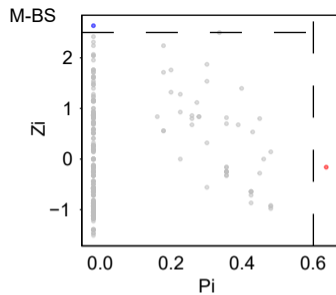

Peripherals

Connectors

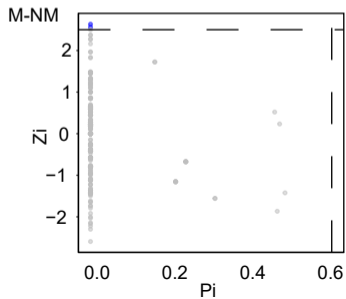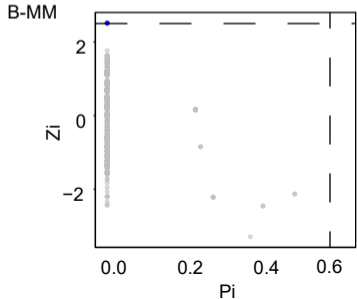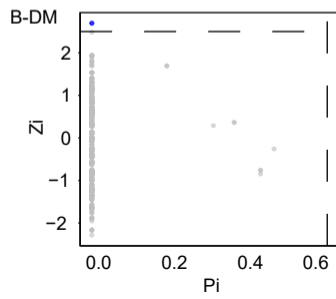

Module hubs

Network hubs

Supplement: Supplementary file 3 [file Image_1.pdf]

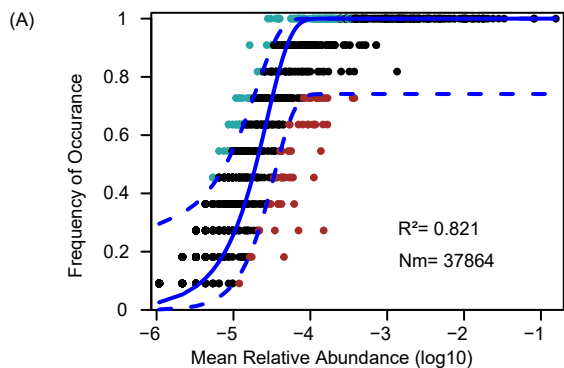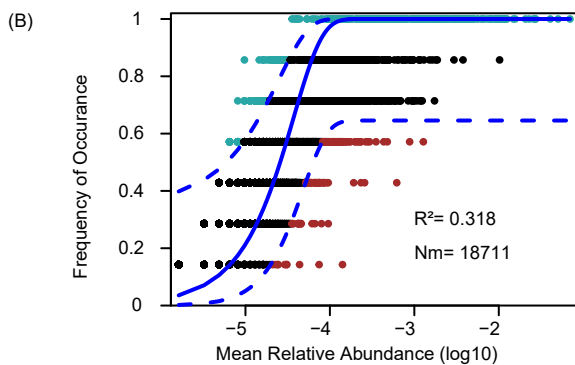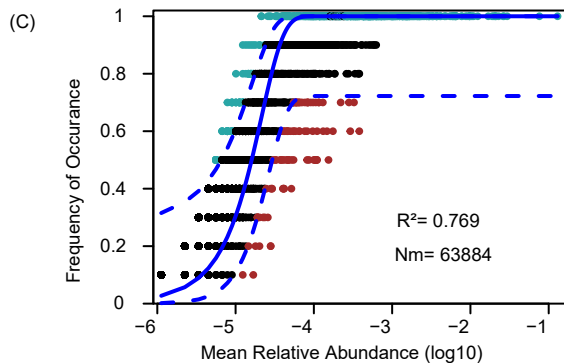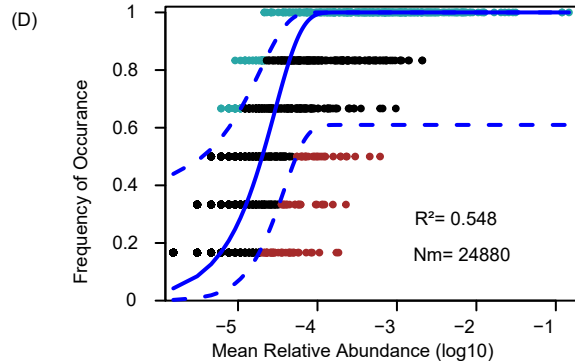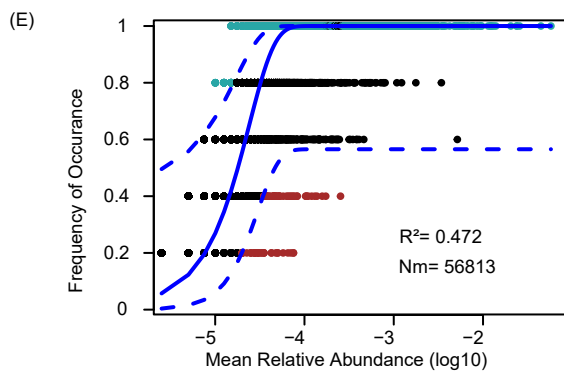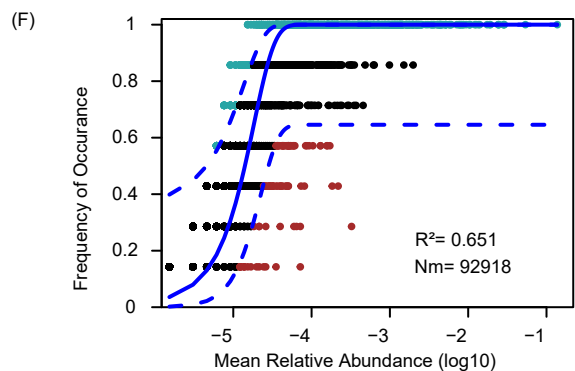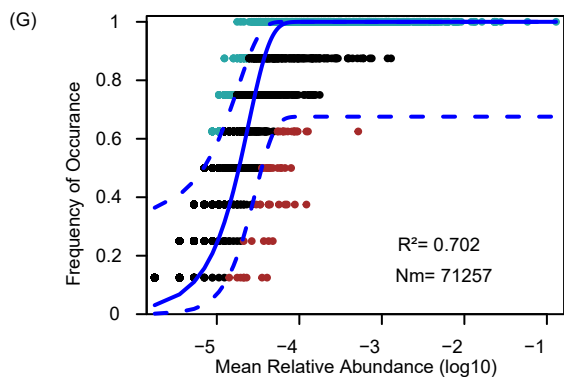

|         |         |
|---------|---------|
| (A)S-BS | (B)S-CW |
| (C)M-BS | (D)M-NM |
| (E)B-CW | (F)B-MM |
| (G)B-DM |         |

Supplement: Supplementary file 4 [file Image_2.pdf]
