# Supplementary material for: Water masses influence the variation of microbial communities in the Yangtze River Estuary and its adjacent waters
Source: Front Microbiol. 2024 Mar 20;15:1367062. doi: 10.3389/fmicb.2024.1367062 (PMC10987813; doi:10.3389/fmicb.2024.1367062)
Supplement: Supplementary file 5 [file Image_3.pdf]

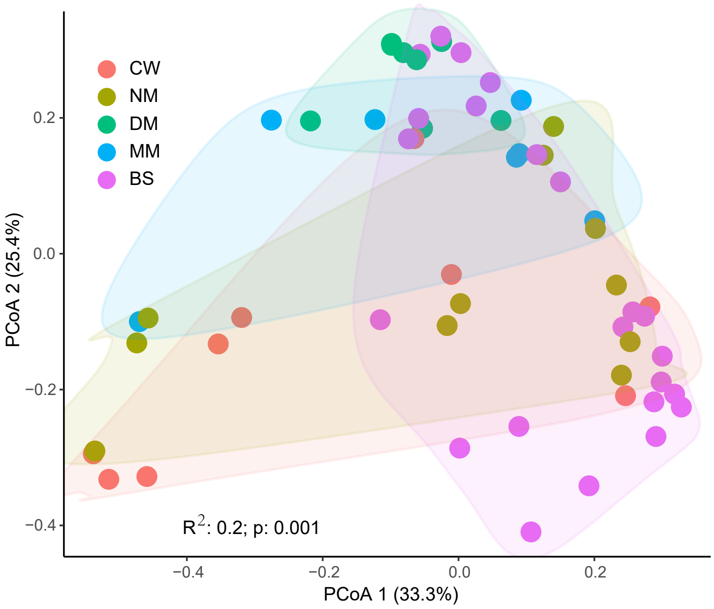

pairs	R^2	p.value	p.adjusted
CW vs NM	0.060	0.275	0.275
CW vs DM	0.302	0.001	0.003
CW vs BS	0.141	0.001	0.003
CW vs MM	0.151	0.062	0.103
NM vs MD	0.206	0.002	0.005
NM vs BS	0.049	0.115	0.144
NM vs MM	0.082	0.169	0.188
DM vs BS	0.175	0.001	0.003
DM vs MM	0.124	0.092	0.131
BS vs MM	0.087	0.022	0.044
